# CCL21/CCR7 axis regulates VEGF-D m^6^A modification to drive lymphangiogenesis and lymphatic metastasis in gallbladder cancer

**DOI:** 10.1007/s13577-026-01420-1

**Published:** 2026-07-24

**Authors:** Zhenheng Wu, Xinran Cai, Lei Jiang, Bin Lin, Wei Pan, Shengzhe Lin, Yanling Chen, Haijie Hong

**Affiliations:** 1 Department of Hepatobiliary Surgery, Fujian Institute of Hepatobiliary Surgery, Fujian Medical University Union Hospital, 29 Xinquan Road, Fuzhou, 350001 Fujian China; 2 Fujian Medical University Cancer Center, Fuzhou, 350122 Fujian China; 3 Key Laboratory of Gastrointestinal Cancer (Fujian Medical University), Ministry of Education, Fujian Medical University, Fuzhou, 350122 Fujian China; 4 Department of Hepatopancreatobiliary Surgery, The First Affiliated Hospital of Fujian Medical University, Fuzhou, 350004 Fujian China; 5 Fujian Center for Drug Evaluation and Monitoring, Fuzhou, 350001 Fujian China

**Keywords:** Gallbladder cancer, VEGF-D, CCL21, Lymphatic metastasis

## Abstract

Gallbladder cancer (GBC), the most common malignancy of the biliary tract, exhibits a high propensity for lymphatic system invasion. However, the mechanisms underlying lymphatic metastasis in GBC remain poorly understood. Here, we demonstrate that C–C motif chemokine ligand 21 (CCL21) is significantly upregulated in GBC and positively correlates with lymphatic vessel density and lymph node metastasis. Both in vivo and in vitro experiments confirm that CCL21/CCR7 axis promotes lymphangiogenesis and lymphatic metastasis in GBC. Mechanistically, CCL21/CCR7 modulates Vascular Endothelial Growth Factor-D (VEGF-D) mRNA N6-Methyladenosine (m^6^A) modification in an AlkB homolog 5 (ALKBH5)-dependent manner, thereby regulating VEGF-D protein expression. Site-directed mutagenesis experiments reveal that m^6^A modification sites in the coding sequence (CDS) region of VEGF-D mRNA are critical for VEGF-D protein regulation. Collectively, our findings highlight that CCL21/CCR7 regulates VEGF-D expression via m^6^A modification, driving lymphatic metastasis in GBC.

**Supplementary Information:**

The online version contains supplementary material available at 10.1007/s13577-026-01420-1.

## Introduction

GBC, the most prevalent form of biliary tract cancer, is a rare yet highly lethal malignancy [1, 2]. The five-year survival rate for GBC patients is a mere 5%, with survival typically not exceeding six months [3]. Surgical resection stands as the curative approach for early-stage GBC. However, the initial stages of GBC are often asymptomatic, leading to diagnoses typically made at advanced stages when surgical intervention is no longer viable [4, 5]. Tumor dissemination and metastasis are significant contributors to mortality in GBC patients, with lymph node metastasis (LNM) being a critical mode of such spread [6]. The early occurrence of lymphatic metastasis is a distinctive feature of GBC and a key factor in its poor prognosis [7] Therefore, identifying the key drivers of high lymphatic metastatic potential in GBC is essential.

Chemokines, upon binding to their receptors, activate downstream pathways that play a pivotal role in various physiological processes including cell growth, development, differentiation, and apoptosis, as well as in the directional migration of lymphocytes, inflammatory cell infiltration, and tumorigenesis [8, 9]. Among these, the CCL21/CCR7 and CXCL12/CXCR4 axes are the most recognized [10]. CCL21 is predominantly expressed in lymphoid tissues or peripheral immune organs, including lymphatic endothelial cells and lymph nodes [11]. Research indicates that C–C motif chemokine receptor 7 (CCR7), a G protein-coupled receptor, serves as the receptor for CCL21 [12]. Recent studies have linked the overexpression of CCR7 to tumor lymphangiogenesis and epithelial-mesenchymal transition-related tumor lymphatic metastasis [13, 14]. This aligns with our previous experimental findings demonstrating CCR7-mediated promotion of lymph node metastasis in GBC through both in vivo and in vitro experiments [15]. However, the functional significance of the chemokine/receptor axis CCL21/CCR7 in GBC remains unreported. Notably, studies have demonstrated that CCL21/CCR7 facilitates lymph node metastasis in esophageal squamous cell carcinoma via ERK1/2 signaling-mediated upregulation of MUC1 [16]. Preclinical data from mouse models suggest that the pro-metastatic capability of the CCL21/CCR7 axis may facilitate the migration of cancer cells from the primary tumor to lymph nodes [17]. Nevertheless, the role of CCL21 in GBC pathogenesis and the mechanistic basis of CCL21/CCR7 axis-mediated lymphatic metastasis remain poorly characterized. To address this knowledge gap, the current study systematically investigates the CCL21/CCR7 axis's regulatory mechanisms in GBC lymphatic metastasis, with particular emphasis on CCL21-mediated signaling pathways.

As a pivotal regulator of lymphangiogenesis, VEGF-D activates downstream signaling pathways (including PI3K/Akt and ERK/MAPK) through binding to the VEGFR-3 receptor. This mechanism directly induces peritumoral lymphatic neogenesis and enhances vascular permeability, thereby establishing a microenvironment conducive to tumor cell invasion into the lymphatic system [18]. Clinically, elevated serum VEGF-D levels in cancer patients demonstrate significant positive correlation with lymph node metastasis and independently associate with reduced overall survival [19, 20]. Beyond its serum expression, heightened VEGF-D levels in tumor tissues have been identified as high-risk drivers of metastatic progression [21, 22]. Nevertheless, the regulatory mechanisms underlying VEGF-D overexpression remain poorly characterized. N6-methyladenosine (m^6^A), the most abundant post-transcriptional modification in eukaryotic mRNA, plays critical roles in regulating biological processes and disease pathogenesis [23]. This dynamic modification is orchestrated by methyltransferase complexes ("writers") and demethylases ("erasers") [24], influencing mRNA stability and consequently modulating target transcript abundance [25]. Emerging evidence highlights m^6^A’s critical involvement in tumor lymphatic metastasis: the m^6^A reader YTHDF1 augments S100P translation to promote lymphatic dissemination [26], while m^6^A-dependent alternative splicing of FOXM1 mediates cervical cancer lymph node metastasis [27]. Intriguingly, VEGF-A—another VEGF family member—undergoes m^6^A modification that accelerates tumor angiogenesis [28]. However, the potential m^6^A modification of VEGF-D itself remains unexplored, and the regulatory impact of this epigenetic mechanism on VEGF-D expression and function has yet to be elucidated.

In this study, we primarily evaluated the impact of the CCL21/CCR7 axis on lymphatic metastasis in GBC and its underlying mechanisms. The results indicate an increased expression of CCL21 in GBC tissues. Both in vivo and in vitro experiments confirmed that CCL21 promotes lymphatic metastasis in GBC in a CCR7-dependent manner. Mechanistically, CCL21/CCR7 facilitates the expression of VEGF-D in GBC cells by inhibiting VEGF-D mRNA m^6^A modification, thereby stimulating human lymphatic endothelial cell sprouting and the formation of new lymphatic vessels.

## Materials and methods

### Patient data collection

In accordance with the protocol approved by the Ethics Committee of the School of Medicine, Fujian Medical University, 65 paraffin-embedded tumor specimens and paired benign tissues from patients clinically and pathologically diagnosed with GBC were collected from the Union Hospital of Fujian Medical University between 2016 and 2024. All patients provided written informed consent. None of the patients had undergone any radiotherapy or chemotherapy prior to tumor resection.

### Cell lines and cell culture

The human GBC cell line NOZ was obtained from the Japanese Collection of Research Bioresources. The human GBC cell line SGC-996 was purchased from the Shanghai Institute of Life Sciences, Chinese Academy of Sciences. The HIBEpiC cell line was acquired from ScienCell Research Laboratories (Carlsbad, CA, USA) and cultured according to the supplier’s instructions. Human dermal lymphatic endothelial cells (HLECs) were acquired from ScienCell Research Laboratories (San Diego, CA, USA). All cell lines were tested for mycoplasma contamination and subjected to short tandem repeat (STR) profiling to confirm their authenticity and purity. HLECs were cultured in endothelial cell medium (ScienCell, San Diego, CA) supplemented with 10% fetal bovine serum (FBS), 1% endothelial cell growth supplement, and 1% antibiotic solution. GBC cells were maintained in DMEM (Hyclone, USA) supplemented with 10% FBS (Hyclone, USA). All cells were incubated at 37 °C in a humidified atmosphere containing 5% CO2. All cell lines were confirmed to be mycoplasma-free.

### Quantitative real-time polymerase chain reaction (RT-qPCR)

Total RNA was extracted from GBC cells using TRIzol reagent (15,596–026, Invitrogen, USA) following the manufacturer’s instructions. mRNA was reverse-transcribed using the All-In-One 5 × RT MasterMix (Abm, Canada). Subsequently, PCR was performed using Fast Start Universal SYBR Green Master Mix (Roche, Basel, Switzerland), and fluorescence was measured using the ABI 7500 Real-Time System (Applied Biosystems, Life Technologies) according to the manufacturer’s protocol. GAPDH was used as an internal control. Data analysis was performed using the 2 − ΔΔCt method. Primer sequences are detailed in Table 1.

**Table 1:** Detailed information of the primer sequences in this study

Gene	Primer sequence
CCL21	F 5′-ATGTGCCTTTGCAACGACTG-3′
	R 5′-TGACAGGGCTTTCCAATGCT-3′
VEGF-D	F 5′-CAGCACATGACGGAGGTTGT-3′
	R 5′-TCATCCAAATACTCCACACGC-3′
GAPDH	F 5′-GGTGTGAACCATGAGAAGTATGA-3′
	R 5′-GAGTCCTTCCACGATACCAAAG-3′
METTL3	F 5′-CTGCAACGCATCATTCGGAC-3′
	R 5′-AGACCCTGGTTGAAGCCTTG-3′
METTL14	F 5′-TGGACCTTGGAAGAGTGTGTT-3′
	R 5′-GTGCTACGCTTCACAGTTCC-3′
WTAP	F 5′-AATCCAGTACCTCAAGCAAGTC-3′
	R 5′-TGTCTTTAGTCTGTTCCAGTTCAC-3′
FTO	F 5′-CTGGAAGCACTGTGGAAGAAG-3′
	R 5′-GCAAGGATGGCAGTCAAGATT-3′
ALKBH5	F 5′-CTCTTCAGCCAGGACGAGTG-3′
	R 5′-CCGTAAGTGTAGCCTTCGCC-3′

### Western blot (WB) and antibodies

Western blot analysis was performed as previously described [29]. Briefly, total protein was extracted from GBC cells using ice-cold RIPA (Radioimmunoprecipitation Assay) buffer (Beyotime, Shanghai, China). Protein concentrations were quantified using the BCA (Bicinchoninic Acid) Protein Assay Kit (Thermo Fisher Scientific). Proteins were separated by SDS-PAGE and transferred to PVDF membranes. After blocking with 5% non-fat milk for 2 h, the membranes were incubated with primary antibodies overnight at 4 °C. Following incubation with secondary antibodies at room temperature for 1 h, antibody-antigen complexes were detected using an ECL kit (Advansta, USA). The following primary antibodies were purchased from Abcam: rabbit anti-CCR7 (ab317369), rabbit anti-ALKBH5 (ab195377), and rabbit anti-VEGF-D (ab155288). Rabbit anti-GAPDH (ab9485) was used as an internal control for normalization. All western blot experiments were independently repeated three times with similar results, and one representative blot from the three independent experiments is shown in the figures. The lanes shown in each western blot panel were cropped from the same blot for presentation purposes, and no lane rearrangement was performed.

### Chemical inhibitors

The following exogenous drugs used in this study were purchased from MCE (MedChemExpress, USA): Actinomycin D (Act-D, HY-17559) and CCL21 (HY-P7166). For GBC cells, Act-D was administered at a concentration of 5 µg/mL, and CCL21 was administered at a concentration of 300 ng/mL. The NF-κB inhibitor Bay 11–7082 (HY-13453), ERK inhibitor PD98059 (HY-12028), JNK inhibitor SP600125 (HY-12041), PI3K/AKT inhibitor LY294002 (HY-10108), and YTHDF2 inhibitor DC-Y13-27 (HY-154919) were purchased from MedChemExpress (MCE, USA). All inhibitors were applied at a final concentration of 20 uM in vitro.

### Immunohistochemical analysis (IHC)

Paraffin-embedded tissue sections were deparaffinized in xylene and rehydrated through a graded ethanol series. Antigen retrieval was performed, and endogenous peroxidase activity was quenched with 3% hydrogen peroxide in TBS for 10 min. Sections were blocked with serum-free blocking reagent and incubated with primary antibodies overnight at 4 °C. Primary antibodies specific for CCL21 (1:200, ab89396), LYVE1 (1:2000, ab219556) and VEGF-D (1:500, ab155288) were used. Secondary antibodies were applied, and sections were incubated at room temperature for 20 min in a humidified chamber. Visualization was achieved using DAB (3,3’-Diaminobenzidine) solution, and sections were counterstained with hematoxylin. Images were captured using a digital slide scanner (Olympus VS200, Japan) and analyzed using Image-Pro Plus 6.0 (Media Cybernetics, USA). Immunohistochemical evaluation was quantified based on the product of staining intensity and the percentage of positively stained cells. The percentage of positively stained cells was scored on a scale of 0 to 4 (0 = 0–10%; 1 = 11–25%; 2 = 26–50%; 3 = 51–75%; 4 = 76–100%), while staining intensity was rated on a scale of 0 to 3 (0 = no staining; 1 = weak; 2 = moderate; 3 = strong). The overall protein expression in each sample was expressed as a histoscore, calculated as the product of the staining intensity score (0–3) and the percentage of positively stained cells score (0–4), ranging from 0 to 12. Low expression was defined as a score of 0–4, and high expression was defined as a score of 6–12. Staining scores were evaluated by two independent pathologists. Same IHC histoscore cutoff (0–4 as low expression and 6–12 as high expression) was consistently applied to CCL21, VEGF-D, and ALKBH5 in all correlation and survival analyses to ensure methodological consistency.

### HLECs tube formation assay

Briefly, Matrigel (BD Biosciences, Cat#356,234, USA) was mixed with serum-free ECM at a 1:2 ratio. The mixture (400 µL) was added to 24-well plates and allowed to solidify at 37 °C overnight. Conditioned media were collected from different GBC cells treatment groups. HLECs (1 × 10^5 cells/well) were seeded onto the Matrigel-coated wells with 300 µL of conditioned media and incubated for 4 h. Tube formation was visualized using an inverted fluorescence microscope (Olympus IX73, Japan), and the total tube length was quantified using ImageJ software (US National Institutes of Health, Bethesda, MD, USA).

### Lymphatic vessel density assessment

Lymphatic vessels were identified by LYVE-1 immunohistochemical staining. Whole-slide scanning at low magnification (40 ×) was performed to identify hotspot regions exhibiting the highest density of LYVE-1-positive lymphatic vessels. Lymphatic vessel density (LVD) was determined by counting LYVE-1-positive lymphatic vessels within these hotspots under high magnification (200 ×). Three independent fields were assessed, and the mean value was calculated. The median measurement served as the cutoff to stratify samples into low and high LVD groups for subsequent statistical analysis.

### mRNA stability

GBC cells were treated with Actinomycin D at a final concentration of 5 µg/mL. After a designated incubation period, RNA was harvested from GBC cells using TRIzol-based extraction and subjected to RT-qPCR analysis.

### Lentiviral constructs and transfection

Lentiviral particles carrying Flag-tagged ALKBH5-GFP (Flag-ALKBH5) and the corresponding control vector were obtained from GENE (Shanghai, China). GBC cells were infected with lentivirus for 24 h, after which the medium was replaced. Stable clones were selected using puromycin (5 µg/mL; Sigma). Lentivector-mediated short-hairpin CCR7 (sh-CCR7) and non-targeting plasmids (sh-Scr) were designed and synthesized by GENE. Stable GBC cell lines with CCR7 knockdown were generated using the same protocol.

### Plasmid transfection

All plasmids for cell transfection were obtained from GENE (Shanghai, China). Cells were seeded in 6-well plates or 10 cm^2^ dishes. When cells reached 70–80% confluence, transfection was performed using Lipofectamine 3000 reagent (Invitrogen, Thermo Fisher Scientific, USA) according to the manufacturer’s instructions. Cells were harvested 48 h post-transfection for subsequent experiments.

### CRISPR–Cas9-mediated CCR7 knockdown

CCR7 knockdown was achieved using a CRISPR-Cas9 system. Single guide RNAs (sgRNAs) targeting human CCR7 were designed and synthesized by GENE (Shanghai, China) and cloned into a lentiviral CRISPR-Cas9 vector. A non-targeting sgRNA was used as a negative control. Lentiviral particles were packaged and produced by GENE (Shanghai, China) according to standard protocols. GBC cells were infected with lentivirus in the presence of polybrene (8 ug/mL) and subsequently selected with puromycin (2 ug/mL) to establish stable CCR7 knockdown cell lines. Knockdown efficiency was validated by Western blot analysis.

### Dual-luciferase reporter assay

GV272 vectors containing the wild-type (WT) or mutant (MUT) VEGF-D CDS region were obtained from GENE (Shanghai, China). WT or MUT VEGF-D CDS plasmids were transfected into different experimental and control groups. After 48 h, firefly luciferase (F-luc) activity was measured using the Dual-Luciferase Reporter Assay System (Promega E2920, USA) according to the manufacturer’s instructions. Renilla luciferase (R-luc) activity was used as an internal control for normalization.

### Popliteal lymph node metastasis model

Female athymic BALB/c nude mice (4–6 weeks old) were purchased from SIPEIFU (Beijing, China). All mice were housed under specific pathogen-free conditions in accordance with the protocol approved by the Ethics Committee of the School of Medicine, Fujian Medical University. All animal experiments were conducted in accordance with the institutional guidelines and national regulations for the care and use of laboratory animals. The study protocol was approved by the Ethics Committee of Fujian Medical University Union Hospital (Approval No. 2021KJCX034). Mice were randomly divided into groups (n = 6 per group). GBC SGC-996 cells (5 × 10^6 cells) suspended in 50 µL of sterile PBS were subcutaneously injected into the left hind footpad of each mouse to establish a footpad-popliteal lymph node metastasis model. For the CCL21-treated group, CCL21 (0.5 µg per injection) was administered intraperitoneally twice weekly. When the footpad tumor volume reached 200 mm^3^, mice were euthanized. Popliteal lymph nodes and footpad primary tumors were collected, paraffin-embedded, and further analyzed by IHC.

### RNA immunoprecipitation (RIP) assay

RIP assays were performed using the Magna RIP RNA-Binding Protein Immunoprecipitation Kit (Millipore, USA) according to the manufacturer’s instructions. Briefly, cells were lysed in RIP lysis buffer supplemented with protease inhibitors and RNase inhibitors. The lysates were incubated with magnetic beads conjugated with antibodies against YTHDF2 (AB220163), IGF2BP1 (AB228741), IGF2BP2 (AB188200), IGF2BP3 (AB220429), or normal rabbit IgG (AB172730) overnight at 4℃ with rotation. After immunoprecipitation, the beads were washed, and RNA–protein complexes were digested with proteinase K to release bound RNA. The RNA was then purified and reverse-transcribed into cDNA, followed by qPCR analysis to detect VEGF-D mRNA enrichment. Relative enrichment was normalized to input RNA and calculated relative to the IgG control.

### Methylated RNA immunoprecipitation (MeRIP-qPCR)

MeRIP-qPCR was performed as previously described [29]. Briefly, 10 µg of total RNA was incubated with affinity beads and an m^6^A antibody in 0.2 mL PCR tubes at room temperature for 90 min using the EpiQuik™ CUT&RUN m^6^A RNA Enrichment Kit (Epigentek, USA). After immunoprecipitation, m^6^A-enriched RNA fragments were eluted, purified, and subjected to reverse transcription followed by RT-qPCR. For MeRIP-qPCR analysis, m^6^A enrichment of VEGF-D mRNA fragments was quantified relative to the corresponding input sample. Specifically, 625 ng of total RNA was used as the input control, and enrichment was calculated as %Input using the formula:

%Input = 100 × 2^(Ct input − Ct IP). IgG immunoprecipitation was performed in parallel as a negative control to account for nonspecific binding. Data from MeRIP-qPCR were analyzed using one-way analysis of variance (ANOVA) followed by Tukey’s multiple comparison test. Primer sequences for VEGF-D m^6^A sites are listed in Table 2.

**Table 2:** Detailed information of the primer sequences in this study

Gene	Primer sequence
VEGF-D 649	F 5’-GATCAGGGCTGCTTCTAGTTTG-3’
	R 5’-TGCTGAGCGAGAGTCCATAC-3’
VEGF-D 700	F 5’-TCACTCTGAGGACTGGAAGC-3’
	R 5’-CAAACCTAGTGGACCGATGG-3’
VEGF-D 792	F 5’- TGCGGCAACTTTCTATGACATT-3’
	R 5’- GTACTCTTCCCCAGCTCACT-3’

### Statistical analysis

All statistical analyses were performed using SPSS 19.0 (SPSS Inc., Chicago, USA) and GraphPad Prism 8 (GraphPad, USA). Differences between two groups were compared using Student’s t-test. Comparisons among multiple groups were performed using one-way ANOVA followed by Tukey’s multiple-comparison test. For mRNA decay assays, relative VEGF-D mRNA expression was measured at different time points after transcriptional inhibition, and differences among treatment groups over time were analyzed using two-way ANOVA followed by multiple-comparison testing. For MeRIP-qPCR assays, m^6^A enrichment was normalized to the corresponding input RNA, and differences among groups at each predicted m^6^A site were analyzed using one-way ANOVA followed by Tukey’s multiple-comparison test. Correlation analyses were performed using Pearson’s or Spearman’s correlation analysis, as appropriate. Kaplan–Meier survival curves were compared using the log-rank test. Cox regression analyses were performed using univariate and multivariate Cox proportional hazards models, and hazard ratios with 95% confidence intervals were reported where applicable. Clinicopathological data were analyzed using the chi-square test or Fisher’s exact test, as appropriate. Lymph node metastasis rates were analyzed as categorical proportion data using Fisher’s exact test based on the raw numbers of metastasis-positive and metastasis-negative mice in each group, rather than using the plotted percentages. Continuous quantitative data are presented as the mean ± standard error of the mean (SEM). A *p*-value < 0.05 was considered statistically significant.

## Results

1. CCL21 is Upregulated in GBC and Correlates with LVD and LNM

**Fig. 1:**
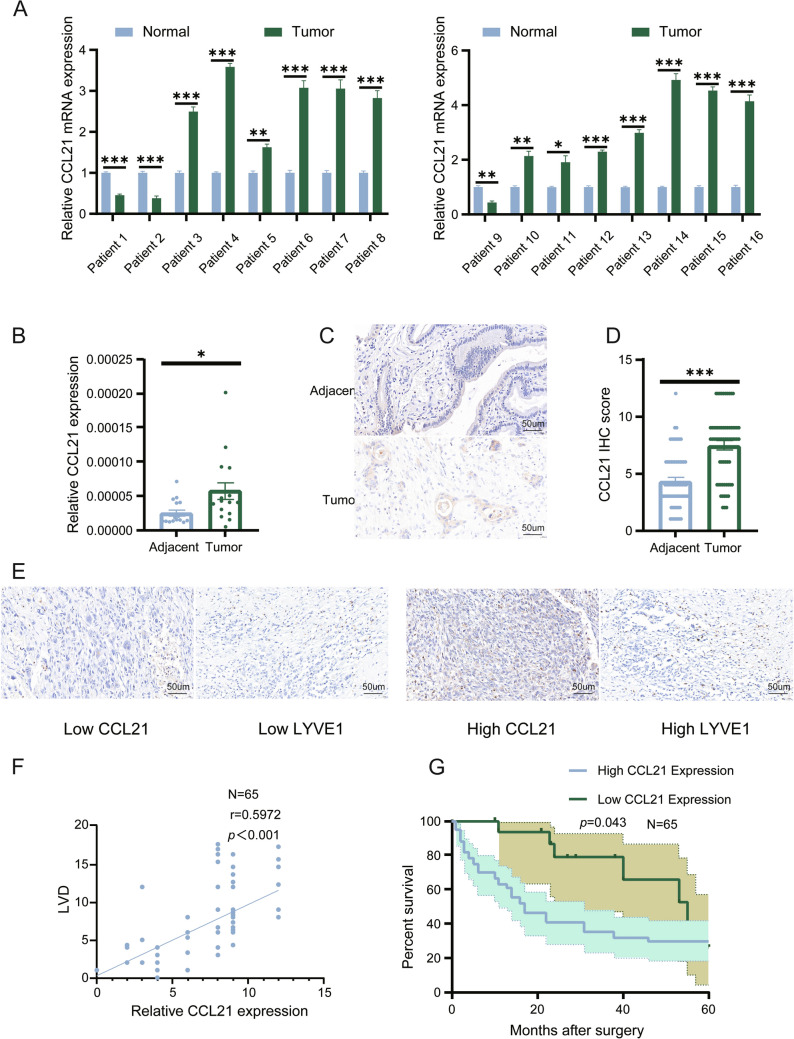
High expression of CCL21 in GBC correlates with LNM and poor prognosis. **A-B** RT-qPCR analysis of CCL21 mRNA expression in 16 pairs of GBC tissues and adjacent non-tumor tissues. **C-D** IHC staining showing CCL21 protein expression in 65 pairs of GBC tissues and adjacent non-tumor tissues. **E** IHC staining showing the expression of CCL21 and LYVE1 proteins in 65 GBC tissues. **F** Pearson correlation analysis of the relationship between CCL21 protein expression and LVD in 65 GBC tissues. **G** Kaplan–Meier survival analysis showing the correlation between CCL21 expression and overall survival in GBC patients. Error bars represent the mean ± SEM. *P < 0.05, **P < 0.01, ***P < 0.001

To elucidate the role of CCL21 in GBC, we first measured CCL21 mRNA expression levels in 16 GBC tumor tissues and paired adjacent tissues using RT-qPCR. The results showed that CCL21 expression was significantly higher in GBC tissues compared to adjacent tissues (Fig. 1A-B). Additionally, IHC staining of 65 GBC tumor tissues and corresponding adjacent tissues revealed elevated CCL21 protein levels in tumor tissues (Fig. 1C-D). CCL21 expression positively correlated with TNM staging including lymph node metastasis but showed no significant association with gender, age, or gallstone presence (Table 3). Furthermore, a significant correlation was also found between CCL21 expression and lymphatic vessel density (LVD) of the GBC tissue (Fig. 1E-F). Kaplan–Meier analysis indicated that GBC patients with low CCL21 expression had a higher overall survival rate compared to those with high expression (Fig. 1G). These findings collectively demonstrate that the oncogene CCL21 is highly expressed in GBC and correlates with lymphangiogenesis and lymphatic metastasis.

**Table 3:** Correlation analysis of CCL21 expression and clinical pathological features in GBC

Clinicopathological features	Case	Protein expression of CCL21		*P*
		High (n = 48)	Low (n = 17)	
Age(years)				0.952
< 60 years old	34	25	9	
≥ 60 years old	31	23	8	
Gender				0.339
Male	28	19	9	
Female	37	29	8	
TNM stage				0.024*
I–II	27	16	11	
III–IV	38	32	6	
Lymph node metastasis				0.019*
( −)	30	18	12	
( +)	35	30	5	
Gallstone				0.422
( +)	29	20	9	
( −)	36	28	8	

Low CCL21 expression is scored 0–4

High CCL21 expression is scored 6–12

Bold value indicates significant differences between groups

^*^P < 0.05

2. CCL21 Regulates GBC Lymphangiogenes and Lymphatic Metastasis via CCR7 *In Vivo* and *In Vitro*

**Fig. 2:**
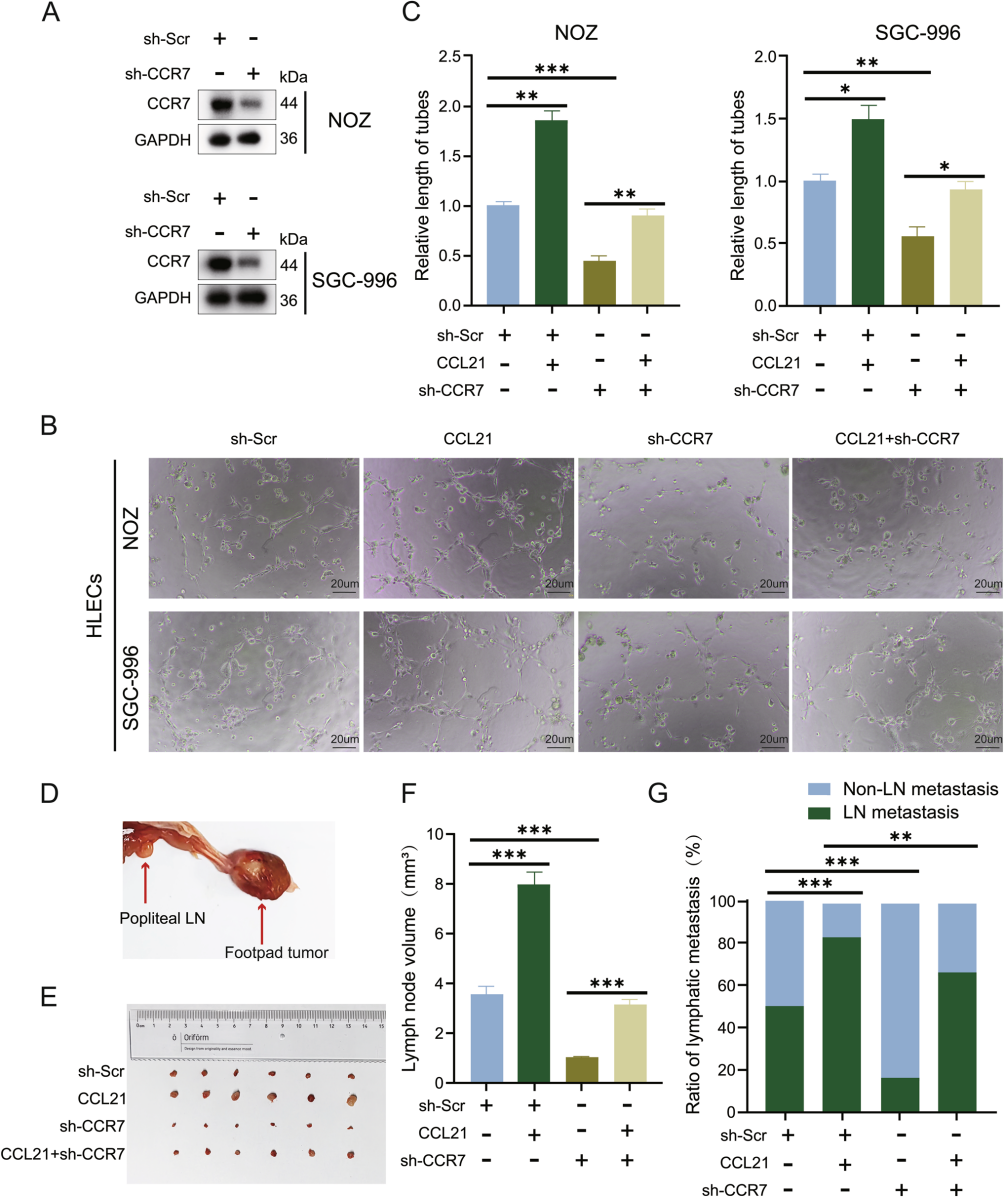
CCL21 regulates GBC LNM via CCR7 in vivo and in vitro. **A** Western blot analysis confirming the knockdown efficiency of sh-CCR7 in GBC cells. **B-C** Lymphatic tube formation assay demonstrating the effect of the CCL21/CCR7 axis on the ability of GBC cells to promote HLEC tube formation. **D** Schematic representation of the successful establishment of a popliteal lymph node metastasis model in nude mice. **E–G** Effects of the CCL21/CCR7 axis on **E** lymph node size, **F** lymph node volume, and **G** lymph node metastasis rate in nude mice. The in vitro experiments, including western blotting and tube-formation assays, were performed with three biological replicates. Six animals were used in the in vivo metastasis model. Continuous quantitative data are presented as the mean ± SEM. Lymph node metastasis rates in panel G were analyzed using Fisher’s exact test. *P < 0.05, **P < 0.01, ***P < 0.001

It is known that CCR7 is a chemokine receptor for CCL21 and the CCL21/CCR7 chemokine-receptor axis plays a pivotal role in tumor lymphatic metastasis [17]. Our previous study has confirmed that CCR7 is related to lymphatic metastasis of GBC [15]. Combined with the above analysis of IHC staining, we speculate that the CCL21/CCR7 axis may promote lymphangiogenesis and lymphatic metastasis of GBC, which is further confirmed in vivo and in vitro. After constructing GBC cells with CCR7 knockdown (sh-CCR7) using lentiviral vectors (Fig. 2A), we performed in vitro lymphatic tube formation assays with HLECs. The results demonstrated that conditioned media from CCL21-stimulated GBC cells significantly enhanced lymphatic tube formation, whereas CCR7 knockdown attenuated this effect (Fig. 2B-C), indicating that CCL21 promotes lymphatic tube formation via CCR7.

To investigate the impact of CCL21/CCR7 on GBC LNM in vivo, we established a footpad-popliteal lymph node metastasis model in mice (Fig. 2D). The results showed that CCL21 significantly increased lymph node volume (Fig. 2E-F) and lymph node metastasis rates (Fig. 2G) compared to the sh-Scr control group. In contrast, sh-CCR7 produced the opposite effects (Fig. 2E-G), consistent with our previous findings. These results confirm that CCL21/CCR7 axis promotes lymphangiogenesis and lymphatic metastasis in GBC.

3. CCL21/CCR7 axis regulates VEGF-D expression in GBC

**Fig. 3:**
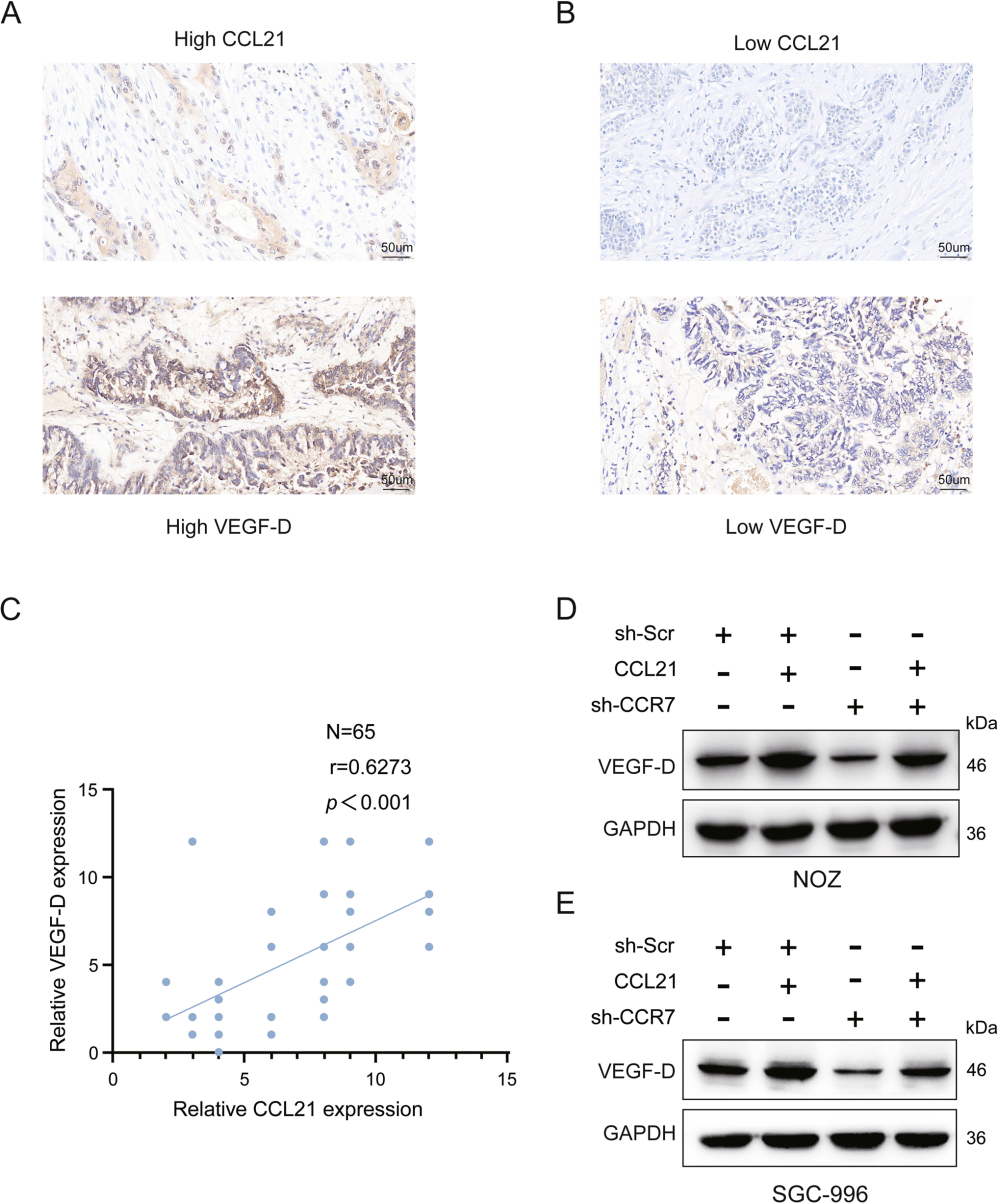
CCL21/CCR7 promotes GBC LNM via VEGF-D. **A-B** IHC staining showing the expression of CCL21 and VEGF-D proteins in 65 GBC tissues. **C** Pearson correlation analysis of the relationship between CCL21 and VEGF-D protein expression in 65 GBC tissues. **D-E** Western blot analysis showing the effect of the CCL21/CCR7 axis on VEGF-D protein expression in GBC cells

VEGF-D is a well-established lymphangiogenic factor [30]. Our prior studies confirmed that VEGF-D is highly expressed in GBC tissues and positively correlates with lymphatic vessel density. Both in vitro and in vivo experiments demonstrated that VEGF-D is involved in GBC-associated lymphangiogenesis [31, 32]. To explore the mechanism of CCL21/CCR7 promoting lymphangiogenesis and lymphatic metastasis in GBC, we examined VEGF-D expression in the 65 GBC patient samples from Fig. 1C-D using IHC. The results showed that high CCL21 expression correlated with high VEGF-D expression (Fig. 3A), and vice versa (Fig. 3B). Correlation analysis confirmed a significant positive relationship between CCL21 and VEGF-D expression (Fig. 3C).Western blot analysis revealed that CCL21 significantly upregulated VEGF-D protein expression, while CCR7 knockdown reversed this effect (Fig. 3D-E), indicating that the CCL21/CCR7 axis regulates VEGF-D expression in GBC. In addition, univariable Cox regression analysis showed that high VEGF-D expression, high CCL21 expression, advanced T classification, and lymph node metastasis were significantly associated with poorer overall survival in patients with GBC, whereas age and gender were not significantly associated with overall survival (Supplementary Fig. S1A). Multivariable Cox regression analysis was then performed by including VEGF-D level, CCL21 level, T classification, lymph node metastasis, age, and gender in the model. After adjustment for these clinicopathological variables, high VEGF-D expression and high CCL21 expression remained significantly associated with poorer overall survival, indicating that VEGF-D and CCL21 are independent prognostic factors for patients with GBC (Supplementary Fig. S1A). Taken together, these findings identify VEGF-D as a critical downstream effector of the CCL21/CCR7 signaling axis.

4. CCL21/CCR7 inhibits m^6^A modification of VEGF-D mRNA in GBC cells

**Fig. 4:**
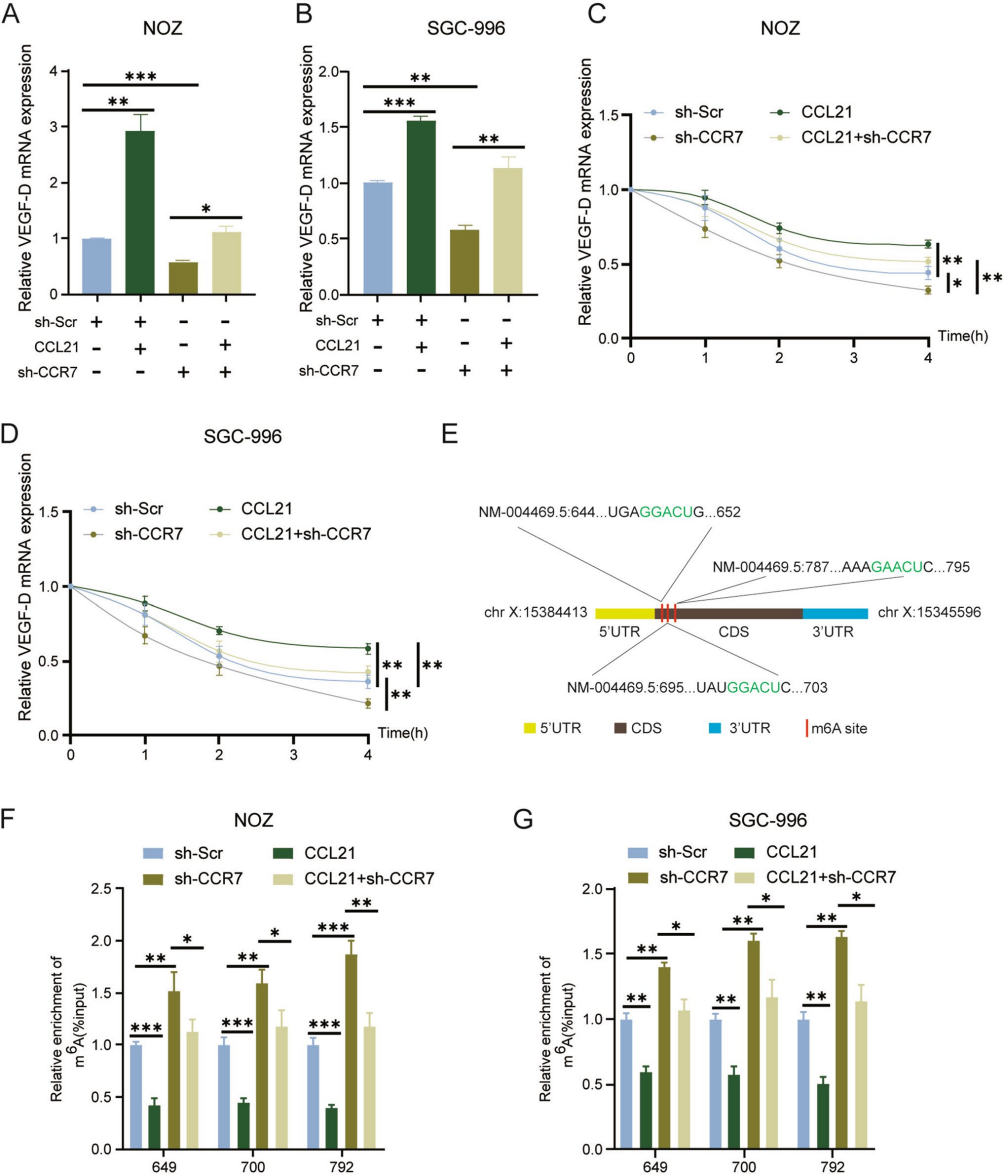
CCL21/CCR7 inhibits m^6^A modification of VEGF-D mRNA in GBC cells. **A-B** RT-qPCR analysis showing the effect of the CCL21/CCR7 axis on VEGF-D mRNA expression in GBC cells. **C-D** RT-qPCR analysis showing the effect of the CCL21/CCR7 axis on VEGF-D mRNA degradation rate in GBC cells. **E** Schematic representation of m^6^A modification sites in the CDS region of VEGF-D mRNA predicted by the SARMP database. **F-G** MeRIP-qPCR analysis showing the effect of the CCL21/CCR7 axis on m.^6^A modification of VEGF-D mRNA in GBC cells. Error bars represent the mean(n = 3) ± SEM. *P < 0.05, **P < 0.01, ***P < 0.001

To further investigate the mechanism by which CCL21/CCR7 regulates VEGF-D, we assessed VEGF-D mRNA expression using qPCR. The results showed that CCL21 significantly upregulated VEGF-D mRNA levels, while CCR7 knockdown attenuated this effect (Fig. 4A-B). RNA stability assays confirmed that CCL21 enhanced VEGF-D mRNA stability, whereas CCR7 knockdown reduced it (Fig. 4C-D), suggesting that CCL21/CCR7 stabilizes VEGF-D mRNA to promote its expression.

In eukaryotes, RNA modifications, particularly m^6^A, play a critical role in RNA stability [33]. Studies have revealed that CCR7 stimulation prevents RNA degradation by removing m^6^A modifications [34]. Therefore, it is plausible that CCL21/CCR7 stabilizes VEGF-D mRNA by modulating its m^6^A modification status. The m^6^A modification sites on VEGF-D mRNA were predicted using the online database SRAMP [35] (http://www.cuilab.cn/ sramp).Within the highest confidence interval, there are three m^6^A methylation sites in the CDS region of VEGF-D mRNA(Fig. 4E). Methylated RNA immunoprecipitation (MeRIP) assays revealed that CCL21 inhibited m^6^A modification in the CDS region of VEGF-D mRNA, while CCR7 knockdown promoted it (Fig. 4F-G). These findings demonstrate that CCL21/CCR7 inhibits m^6^A modification of VEGF-D mRNA in GBC cells.

5CCL21/CCR7 inhibits VEGF-D mRNA m^6^A modification via ALKBH5 in GBC cells

**Fig. 5:**
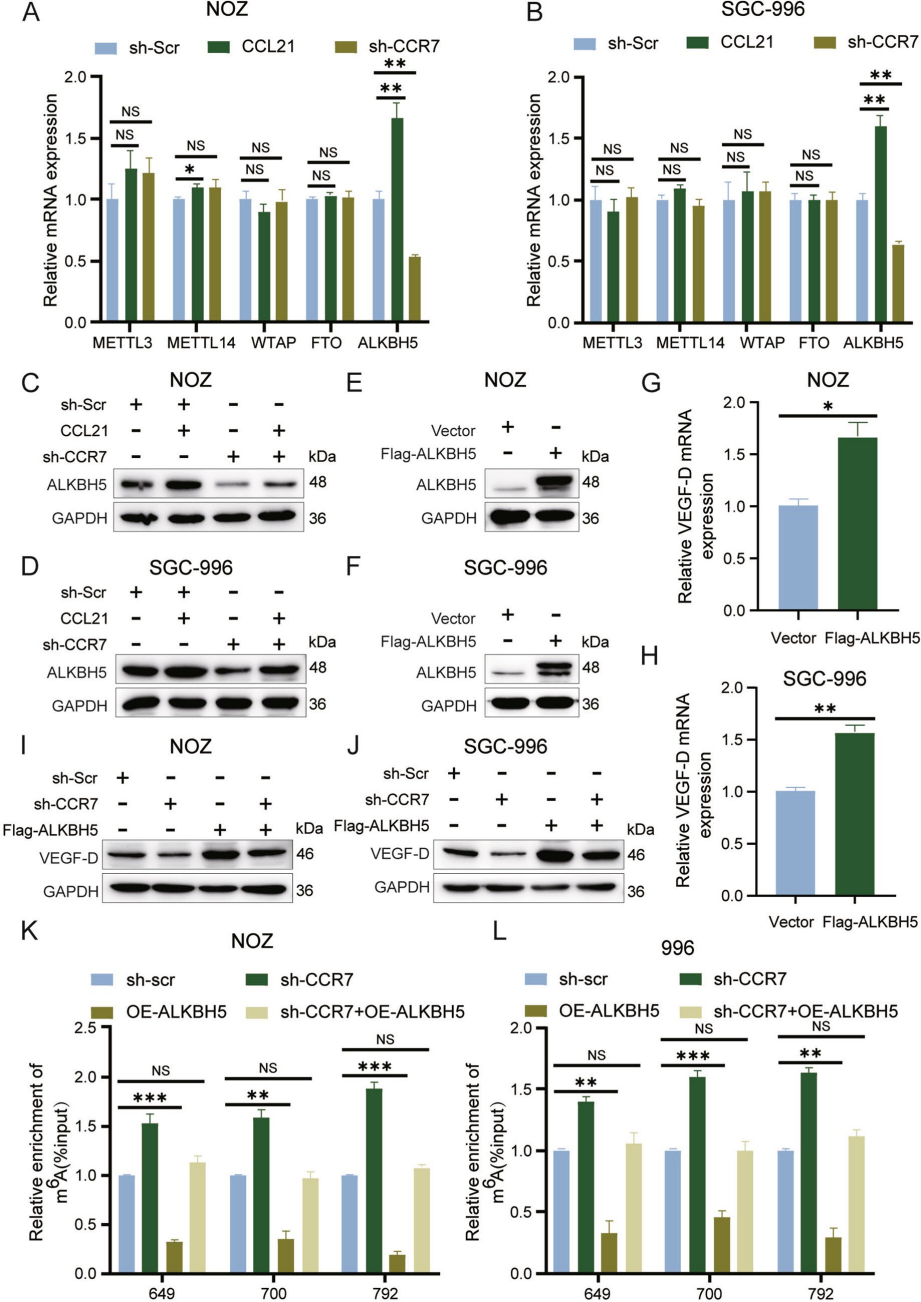
CCL21/CCR7 inhibits m^6^A modification of VEGF-D mRNA via ALKBH5 in GBC cells. **A-B** RT-qPCR analysis showing the effect of the CCL21/CCR7 axis on the mRNA expression of key m^6^A modification-related molecules in GBC cells. **C-D** Western blot analysis showing the effect of the CCL21/CCR7 axis on ALKBH5 protein expression in GBC cells. **E–F** Western blot analysis confirming the overexpression efficiency of Flag-ALKBH5 in GBC cells. **G-H** RT-qPCR analysis showing the effect of Flag-ALKBH5 on VEGF-D mRNA expression in GBC cells. **I-J** Western blot analysis showing the rescue effect of Flag-ALKBH5 on VEGF-D protein expression after CCR7 knockdown. **K-L** MeRIP-qPCR analysis showing the rescue effect of Flag-ALKBH5 on m.^6^A modification of VEGF-D mRNA after CCR7 knockdown. Error bars represent the mean(n = 3) ± SEM. *P < 0.05, **P < 0.01, ***P < 0.001

Following CCL21/CCR7 stimulation, we examined changes in key components of the m^6^A methyltransferase complex (METTL3/METTL14/WTAP) and demethylases (ALKBH5 and FTO) using RT-qPCR and Western blot [36]. RT-qPCR showed that CCL21 significantly upregulated ALKBH5 mRNA levels, while CCR7 knockdown suppressed them (Fig. 5A-B). Western blot analysis confirmed that CCL21 enhanced ALKBH5 protein expression, whereas CCR7 knockdown reduced it (Fig. 5C-D). To minimize potential off-target effects associated with shRNA-mediated CCR7 knockdown, we employed a CRISPR–Cas9-based gene editing approach to silence CCR7 (CCR7-KD) (Supplementary Fig. S2A). Consistent with the shRNA results, CCR7-KD markedly attenuated the CCL21-induced upregulation of ALKBH5 protein expression, further supporting a critical role of CCR7 in mediating this effect (Supplementary Fig. S2B). CCR7 is known to activate multiple downstream signaling pathways, including ERK, PI3K/AKT, JNK, and NF-κB [37, 38]. To investigate whether these pathways are involved in CCL21/CCR7-mediated regulation of ALKBH5 expression, specific pharmacological inhibitors were applied. Notably, treatment with the NF-κB inhibitor Bay 11–7082 markedly attenuated the CCL21-induced increase in ALKBH5 mRNA expression, whereas inhibition of other pathways showed no significant effect (Supplementary Fig. S2C). Collectively, suggesting that CCL21/CCR7 inhibits VEGF-D mRNA m^6^A modification by upregulating ALKBH5.

To further explore the role of ALKBH5, we established GBC cells stably overexpressing ALKBH5 (Flag-ALKBH5) using lentiviral constructs (Fig. 5E-F). RT-qPCR and Western blot results demonstrated that ALKBH5 overexpression promoted VEGF-D mRNA and protein expression and counteracted the effects of CCR7 knockdown (Fig. 5G-J). MeRIP assays confirmed that ALKBH5 inhibited VEGF-D mRNA m^6^A modification and reversed the effects of CCR7 knockdown (Fig. 5K-L). These findings indicate that ALKBH5 mediates the CCL21/CCR7-dependent inhibition of VEGF-D mRNA m^6^A modification.

m^6^A reader proteins, including YTHDF2 and IGF2BPs, are known to regulate the stability of m^6^A-modified mRNAs [39]. To identify the reader involved in CCL21/CCR7-mediated regulation of VEGF-D mRNA stability, RIP-qPCR assays were performed. The results showed that CCL21 stimulation decreased the binding of YTHDF2 to VEGF-D mRNA, whereas sh-CCR7 led to increased YTHDF2 association (Supplementary Fig. S2D-E). In contrast, no significant changes were observed in the binding of IGF2BPs to VEGF-D mRNA upon modulation of CCL21/CCR7 signaling (Supplementary Fig. S2D-E). Furthermore, treatment with the YTHDF2 inhibitor DC-Y13-27 increased VEGF-D mRNA stability in CCR7-knockdown cells (Supplementary Fig. S2F-G). In addition, siRNA-mediated knockdown of YTHDF2 (Supplementary Fig. S2H) further confirmed its functional role. Specifically, inhibition of YTHDF2 partially rescued the sh-CCR7-induced reduction in VEGF-D mRNA stability (Supplementary Fig. S2I). These results suggest a potential involvement of m^6^A reader YTHDF2 in this process.

6CCL21/CCR7 and ALKBH5 regulate VEGF-D expression via m^6^A modification sites

**Fig. 6:**
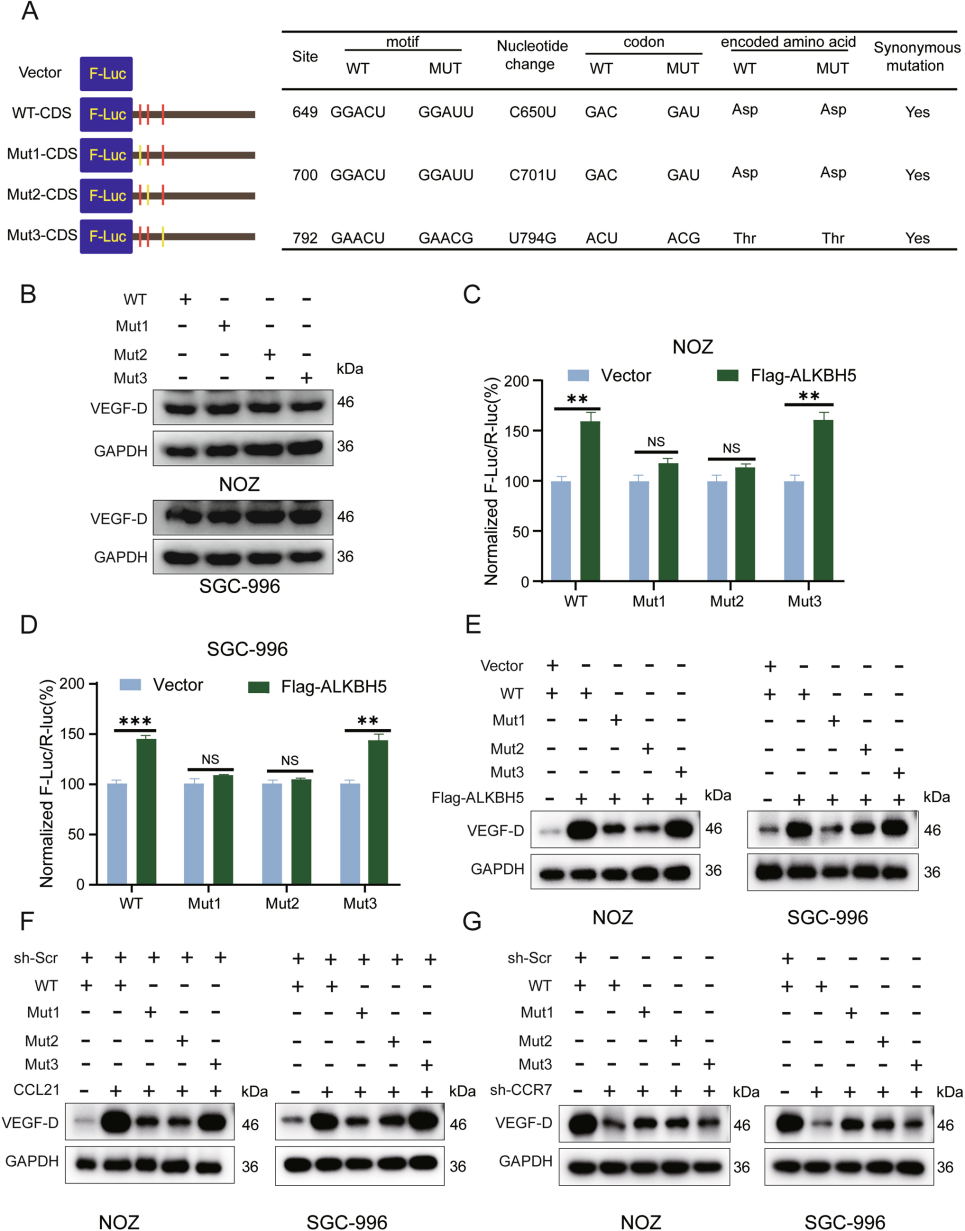
CCL21/CCR7 and ALKBH5 regulate VEGF-D expression via m^6^A modification sites in VEGF-D mRNA. **A** Schematic representation of the construction of wild-type VEGF-D CDS plasmid (WT) and three synonymous motif-mutant plasmids (Mut1, Mut2, and Mut3) targeting predicted m.^6^A motifs within the CDS region of VEGF-D mRNA. The introduced substitutions disrupted the RRACH motif context while preserving the encoded VEGF-D amino acids. **B** Western blot analysis of VEGF-D protein expression in GBC cells transfected with WT or MUT plasmids. **C-D** Dual-luciferase reporter assay showing the effect of Flag-ALKBH5 on luciferase activity in GBC cells transfected with WT or MUT plasmids. **E** Western blot analysis showing the effect of Flag-ALKBH5 on VEGF-D protein expression in GBC cells transfected with WT or MUT plasmids. **F** Western blot analysis showing the effect of CCL21 on VEGF-D protein expression in GBC cells transfected with WT or MUT plasmids under CCL21 stimulation. **G** Western blot analysis showing the effect of sh-CCR7 on VEGF-D protein expression in GBC cells transfected with WT or MUT plasmids. Error bars represent the mean(n = 3) ± SEM. **P < 0.01, ***P < 0.001

To determine whether the predicted m^6^A motifs within the VEGF-D mRNA CDS region are involved in VEGF-D regulation, we constructed a wild-type CDS plasmid (WT-CDS) and three synonymous motif-mutant plasmids (Mut1-CDS, Mut2-CDS, and Mut3-CDS) (Fig. 6A). In these mutant constructs, nucleotides adjacent to the predicted m^6^A-modified adenosines within the RRACH motifs were substituted to disrupt the m^6^A consensus motif while preserving the encoded VEGF-D amino acids. Specifically, the introduced substitutions were C650U, C701U, and U794G at the mRNA level, corresponding to GAC-to-GAU, GAC-to-GAU, and ACU-to-ACG codon changes, respectively (Fig. 6A). Western blot analysis showed comparable VEGF-D protein levels between the WT-CDS and mutant constructs in both NOZ and SGC-996 cells, suggesting that these synonymous motif mutations did not markedly affect VEGF-D protein expression (Fig. 6B). Dual-luciferase reporter assays showed that ALKBH5 significantly enhanced the fluorescence intensity of WT-CDS and Mut3-CDS but had minimal impact on Mut1-CDS and Mut2-CDS (Fig. 6C-D). Western blot analysis confirmed that ALKBH5 promoted VEGF-D protein expression via WT-CDS and Mut3-CDS but not Mut1-CDS and Mut2-CDS (Fig. 6E), indicating that ALKBH5 regulates VEGF-D expression by targeting m^6^A modification sites 649 and 700 in the VEGF-D mRNA CDS region.

Similarly, CCL21 promoted VEGF-D protein expression via WT-CDS and Mut3-CDS but not Mut1-CDS and Mut2-CDS (Fig. 6F). Conversely, CCR7 knockdown inhibited VEGF-D protein expression via WT-CDS and Mut3-CDS but not Mut1-CDS and Mut2-CDS (Fig. 6G). These results demonstrate that CCL21/CCR7 promotes VEGF-D protein expression by inhibiting m^6^A modification at sites 649 and 700 in the VEGF-D mRNA CDS region.

7ALKBH5 promotes lymphatic metastasis and is clinically associated with CCL21/CCR7 signaling in GBC

To further evaluate the role of ALKBH5 in CCL21/CCR7-mediated lymphatic metastasis in vivo, rescue experiments were performed. Flag-ALKBH5 significantly increased lymph node volume and the incidence of lymphatic metastasis (Supplementary Fig. S3A-C). In addition, qPCR analysis showed that the mRNA expression levels of CCL21, ALKBH5, and VEGF-D were elevated in GBC cells (Supplementary Fig. S3D). Consistently, clinical data revealed a positive correlation between CCL21 and ALKBH5 expression in GBC tissues (Supplementary Fig. S3E). Moreover, ALKBH5 expression was higher in patients with lymph node metastasis (Supplementary Fig. S3F), and elevated ALKBH5 expression was associated with shorter overall survival (Supplementary Fig. S3G). Taken together, these findings support a role for ALKBH5 in mediating CCL21/CCR7-driven lymphatic metastasis in GBC.

Collectively, these findings establish a mechanistic link between CCL21/CCR7 signaling and epitranscriptomic regulation in GBC. Specifically, activation of the CCL21/CCR7 axis activates NF-κB signaling, which is involved in the induction of ALKBH5 expression, leading to reduced m^6^A modification of VEGF-D mRNA at critical CDS sites. The resulting decrease in YTHDF2-mediated mRNA decay enhances VEGF-D mRNA stability and protein expression, thereby promoting lymphangiogenesis and lymphatic metastasis in GBC (Supplementary Fig. S4). These results identify the CCL21/CCR7–ALKBH5–VEGF-D signaling axis as a key regulatory pathway driving lymphatic dissemination in GBC.

## Discussion

In recent years, accumulating evidence has highlighted the significance of the CCL21/CCR7 axis in the distant metastasis of various cancers [10, 40]. This axis has emerged as a key target for controlling tumor metastasis [41]. The high propensity for distant metastasis is a hallmark of the aggressive nature of GBC [42]. Therefore, a deeper understanding of the role and mechanisms of CCL21 in GBC could provide valuable insights for the development of novel therapeutic strategies. In this study, we demonstrated that CCL21 is upregulated in GBC and its expression correlates with lymphatic vessel density and lymph node metastasis in GBC patients. Both in vitro and in vivo experiments confirmed that CCL21 promotes lymphangiogenesis and lymphatic metastasis of GBC in a CCR7-dependent manner. These results suggest that the CCL21/CCR7 axis may be a key driver of lymphatic metastasis in GBC. A limitation of this study is that the precise cellular source of CCL21 in gallbladder cancer tissues was not experimentally determined. Although CCL21 is generally considered to be produced by lymphatic endothelial cells and lymphoid stromal compartments, our current data cannot distinguish whether CCL21 in tumor tissues is derived from tumor cells, stromal cells, lymphatic endothelial cells, or multiple cellular components within the tumor microenvironment. Future studies using spatial localization approaches or double immunostaining will be required to more precisely define the cellular origin of CCL21 in gallbladder cancer.

VEGF-D-mediated lymphangiogenesis is a crucial step in the regional lymph node dissemination of GBC [43]. However, the mechanisms driving high VEGF-D expression in GBC remain poorly understood. In this study, we demonstrated that the CCL21/CCR7 axis is a key factor driving VEGF-D expression in GBC. Further investigations revealed that the CCL21/CCR7 axis enhances VEGF-D mRNA stability, thereby regulating VEGF-D protein expression. A potential limitation of this study is the relatively small sample size, which may introduce a risk of overfitting in the multivariable Cox regression model. Therefore, the prognostic findings should be interpreted with caution and validated in larger independent cohorts. Studies have shown that m^6^A modification dynamically regulates mRNA stability and protein expression levels [44]. For instance, the m^6^A reader protein YTHDF2 negatively impacts mRNA stability [45], while IGF2BPs positively regulate mRNA stability [46]. To date, there have been no reports on m^6^A modification of VEGF-D mRNA. In this study, MeRIP-qPCR experiments confirmed that CCL21 inhibits m^6^A modification in the CDS region of VEGF-D mRNA, whereas sh-CCR7 promotes it. Collectively, these findings suggest that the CCL21/CCR7 axis regulates VEGF-D protein expression in GBC by modulating m^6^A modification in the CDS region of VEGF-D mRNA.

In recent years, m^6^A modification has garnered significant attention in GBC research. In GBC, IGF2BP3 enhances GBC invasiveness by stabilizing KLK5 mRNA in an m^6^A-dependent manner, activating the PAR2/AKT axis [47]. Another study demonstrated that ALKBH5 mediates TGF-β1-induced m^6^A modification of FOXA1 mRNA, influencing GBC invasion and migration [29]. In this study, we examined the effects of the CCL21/CCR7 axis on the expression of key components of the m^6^A methyltransferase complex (METTL3/METTL14/WTAP) and demethylases (ALKBH5 and FTO) in GBC cells. To investigate whether classical CCR7 downstream signaling pathways are involved in the regulation of ALKBH5 expression, pharmacological inhibitors targeting major signaling cascades were applied. Notably, inhibition of NF-κB signaling using Bay 11–7082 significantly reduced CCL21-induced ALKBH5 mRNA upregulation, whereas blockade of other pathways showed no significant effect. These findings suggest that NF-κB signaling is involved in, and may contribute to, CCL21/CCR7-mediated ALKBH5 induction, although this does not establish a direct transcriptional regulatory relationship. Our results suggest that the CCL21/CCR7 axis may inhibit m^6^A modification of VEGF-D mRNA by upregulating the expression of the demethylase ALKBH5, consistent with the reported role of CCR7 in removing m^6^A modifications [34]. Site-directed mutagenesis experiments revealed that ALKBH5 promotes VEGF-D protein expression by inhibiting m^6^A modification at sites 649 and 700 in the CDS region of VEGF-D mRNA. Interestingly, the CCL21/CCR7 axis also regulates VEGF-D protein expression in a manner dependent on these two m^6^A modification sites, further elucidating a novel mechanism of VEGF-D regulation in GBC.

While the role of the CCL21/CCR7 axis in lymphatic metastasis has been extensively reported, our findings extend this paradigm by uncovering an epitranscriptomic layer of regulation. Specifically, we identify an ALKBH5-dependent m^6^A modification mechanism that governs VEGF-D expression downstream of CCR7 signaling, thereby linking chemokine signaling to RNA modification in lymphangiogenesis. However, our current study focuses primarily on VEGF-D, and whether CCR7 signaling broadly regulates additional m^6^A-modified transcripts remains to be determined. Beyond the mechanistic insights, our study also provides a potential framework for therapeutic intervention. The identification of the CCL21/CCR7–ALKBH5–VEGF-D axis highlights several nodes that may be amenable to targeting. In particular, inhibition of CCR7 signaling or modulation of m^6^A regulators such as ALKBH5 could represent promising strategies to suppress lymphangiogenesis and lymphatic metastasis in GBC. Notably, epitranscriptomic regulation of pro-lymphangiogenic factors may offer an additional layer of therapeutic control beyond conventional transcriptional targeting. While the present study does not directly evaluate pharmacological interventions, future investigations exploring inhibitors of CCR7 or modulators of m^6^A machinery will be important to translate these findings into clinical applications. Although our in vivo rescue experiments demonstrate that ALKBH5 functions as a critical downstream effector of CCR7 signaling, the present study does not fully establish VEGF-D as the sole indispensable mediator of ALKBH5-driven lymphatic metastasis in vivo. Given the broad transcriptome-wide regulatory role of ALKBH5, it is possible that additional m^6^A-modified transcripts may also contribute to its pro-lymphangiogenic effects. Therefore, VEGF-D should be considered a key but not exclusive downstream target in this regulatory axis.

While our study establishes a CCL21/CCR7–ALKBH5–VEGF-D regulatory axis in lymphatic metastasis, it also raises several mechanistic and translational questions that warrant further investigation. First, although VEGF-D is identified as a key downstream effector, m^6^A-dependent regulation is unlikely to be limited to a single transcript. Given the widespread impact of m^6^A on RNA metabolism, it is plausible that additional lymphangiogenic or metastasis-related factors are coordinately regulated within this framework. Systematic profiling of m^6^A-modified transcripts under CCL21/CCR7 activation may therefore reveal a broader regulatory network. Second, our findings implicate ALKBH5 as a critical mediator linking chemokine signaling to epitranscriptomic regulation, raising the possibility that this axis could be therapeutically targeted. Modulation of CCR7 signaling or ALKBH5 activity may represent complementary strategies to interfere with lymphatic dissemination. However, given the context-dependent roles of m^6^A regulators, careful evaluation of specificity and potential systemic effects will be required. Finally, considering that lymphatic metastasis is a common feature across multiple malignancies, it will be of interest to determine whether similar signaling-m^6^A regulatory coupling exists in other cancer types with high lymphatic tropism. Elucidating such conserved mechanisms may broaden the biological and clinical relevance of our findings.

## Supplementary Information

Below is the link to the electronic supplementary material.

Supplementary file1 (DOCX 3781 KB)

Figure S1: CCL21/CCR7 promotes GBC LNM via VEGF-D. (A) The upper panel shows the univariable and multivariable Cox regression analyses of factors associated with overall survival in patients with GBC, including comparison groups, hazard ratios (HRs), 95% confidence intervals (CIs), and exact P values. The lower panel shows the forest plot of the multivariable Cox regression analysis. The Cox regression analysis included 65 patients with available survival data, including 47 death events. Variables entered into the multivariable model included VEGF-D level, CCL21 level, T classification, lymph node metastasis, age, and gender. Bold values indicate P < 0.05. Supplementary file2 (TIF 2426 KB)

Figure S2: CCL21/CCR7 inhibits m6A modification of VEGF-D mRNA via ALKBH5 in GBC cells. (A) Validation of CCR7 knockdown efficiency by CRISPR–Cas9 (CCR7-KD). (B) Rescue experiments showing the effect of CCR7-KD on CCL21-mediated ALKBH5 expression by Western blot. (C) RT-qPCR analysis of the inhibitory effects of pathway inhibitors on CCL21-induced ALKBH5 mRNA expression. (D-E) RIP-qPCR assays demonstrating the binding of YTHDF2 and IGF2BPs to VEGF-D mRNA. (F-G) YTHDF2 inhibitor treatment increased VEGF-D mRNA stability in CCR7-knockdown cells. (H) Western blot analysis confirming the knockdown efficiency of si-YTHDF2 in GBC cells. (I) Knockdown of YTHDF2 by siRNA partially rescued the decrease in VEGF-D mRNA stability induced by CCR7 knockdown. Error bars represent the mean（n=3）±SEM. **P＜0.01, ***P＜0.001. Supplementary file3 (TIF 5050 KB)

Figure S3: ALKBH5 promotes lymphatic metastasis and is clinically associated with CCL21/CCR7 signaling in GBC. (A-C) Effects of Flag-ALKBH5 overexpression on CCL21/CCR7 axis-mediated lymphatic metastasis in nude mice. (A) Lymph node size, (B) lymph node volume, and (C) lymph node metastasis rate. (D) mRNA expression levels of CCL21, ALKBH5, and VEGF-D in normal biliary epithelial cells and GBC cell lines. (E) Correlation analysis of CCL21 and ALKBH5 expression in GBC tissues based on IHC from 65 patients. (F) Comparison of ALKBH5 expression between GBC patients with and without lymph node metastasis. (G) Kaplan-Meier survival analysis of GBC patients stratified by ALKBH5 expression. Error bars represent the mean（n=3）±SEM. **P＜0.01, ***P＜0.001. Supplementary file4 (TIF 1771 KB)

Figure S4: Proposed model illustrating the CCL21/CCR7–ALKBH5–VEGF-D signaling axis in gallbladder cancer lymphatic metastasis. Activation of the CCL21/CCR7 axis induces NF-κB signaling and upregulates ALKBH5 expression. ALKBH5 decreases m6A modification of VEGF-D mRNA at key CDS sites, reducing YTHDF2-dependent mRNA degradation and increasing VEGF-D mRNA stability and protein expression. Elevated VEGF-D promotes lymphangiogenesis and lymphatic vessel permeability, facilitating lymphatic dissemination and lymph node metastasis of gallbladder cancer cells. Supplementary file5 (TIF 54479 KB)

## Data Availability

The datasets used and analyzed during the current study are available from the corresponding author on reasonable request.
