# Supplementary figures and images for: CCL21/CCR7 axis regulates VEGF-D m6A modification to drive lymphangiogenesis and lymphatic metastasis in gallbladder cancer
Source: Hum Cell. 2026 Jul 24;39(8):114. doi: 10.1007/s13577-026-01420-1 (PMC13400605; doi:10.1007/s13577-026-01420-1)

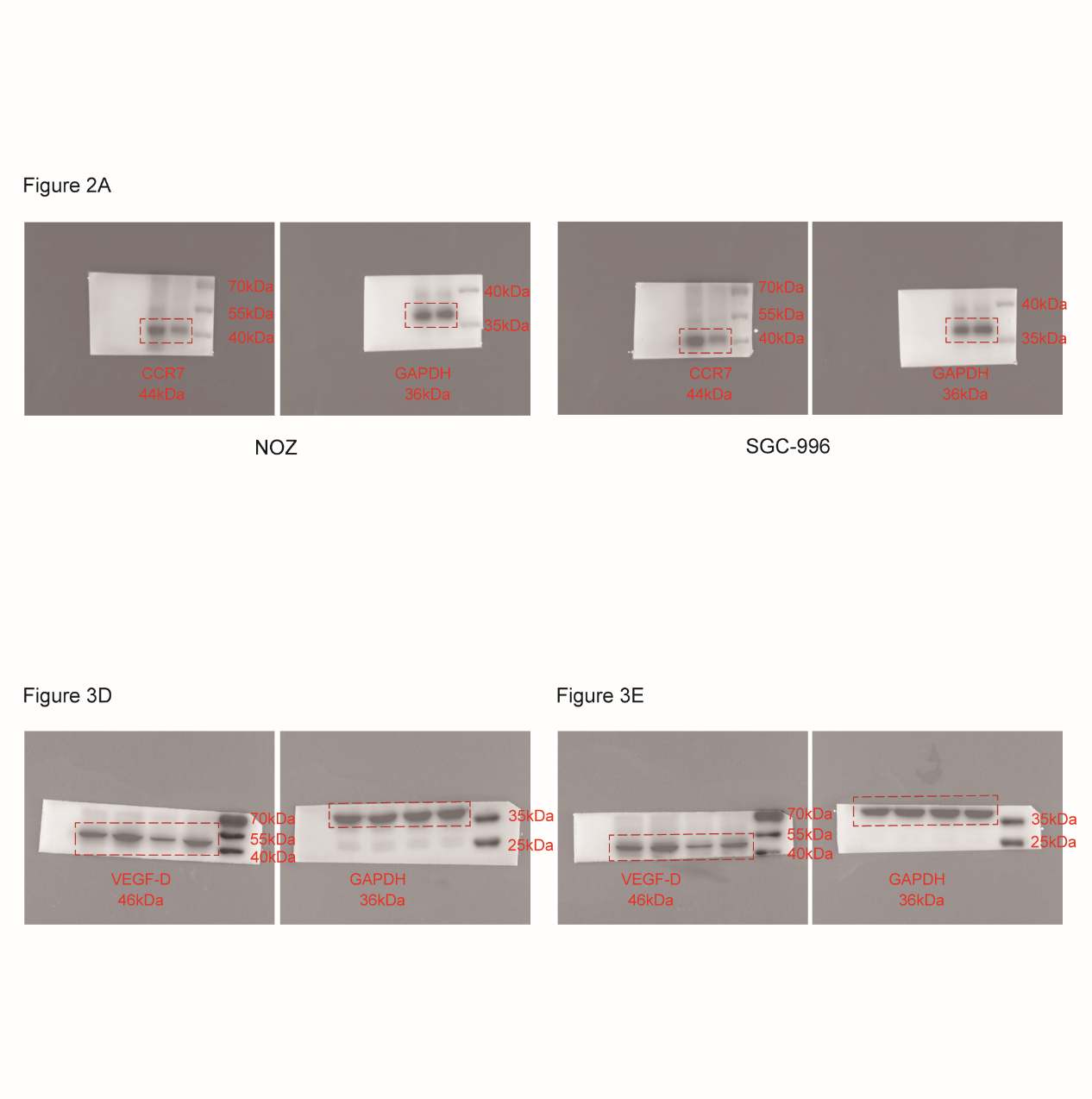


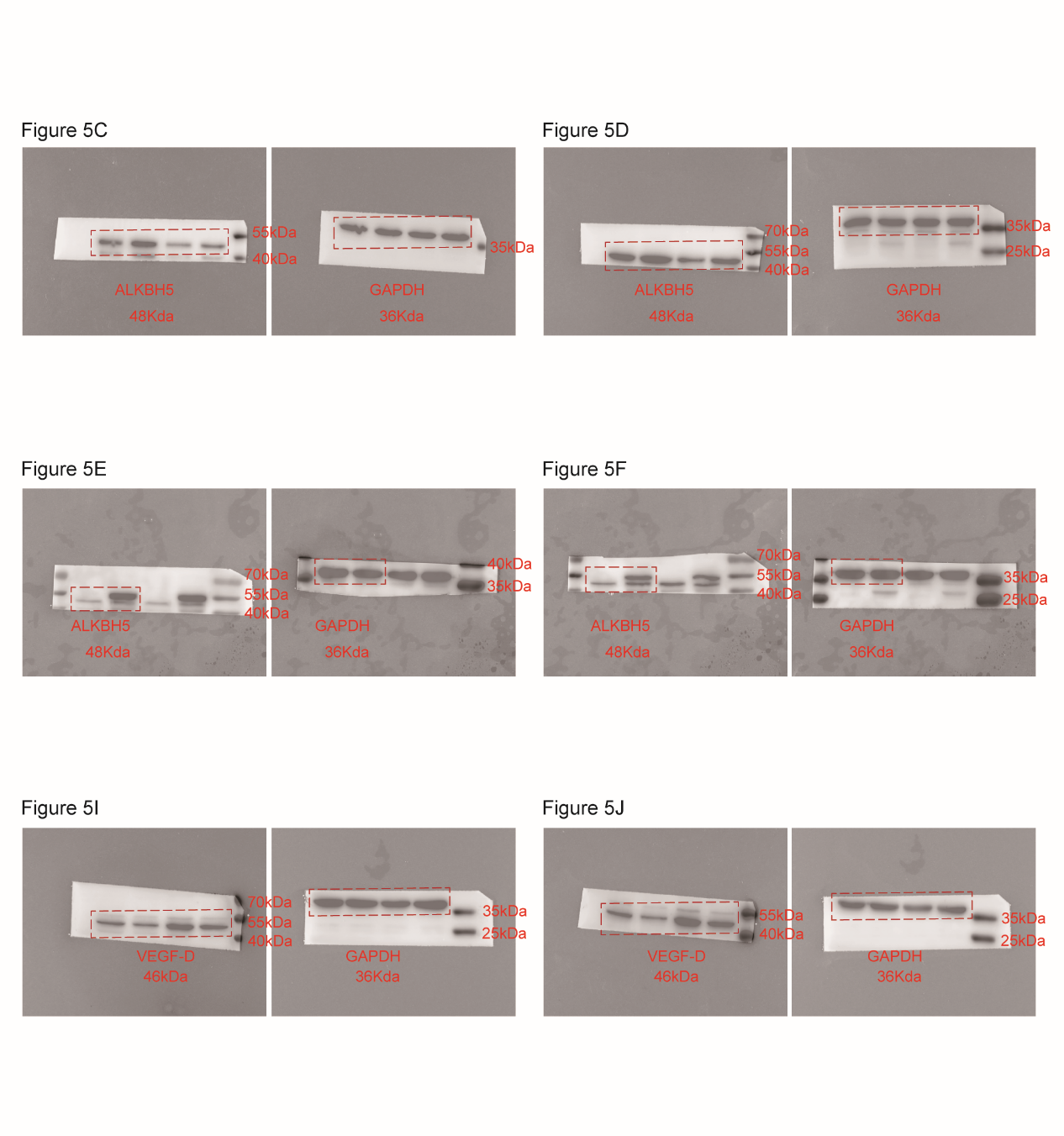


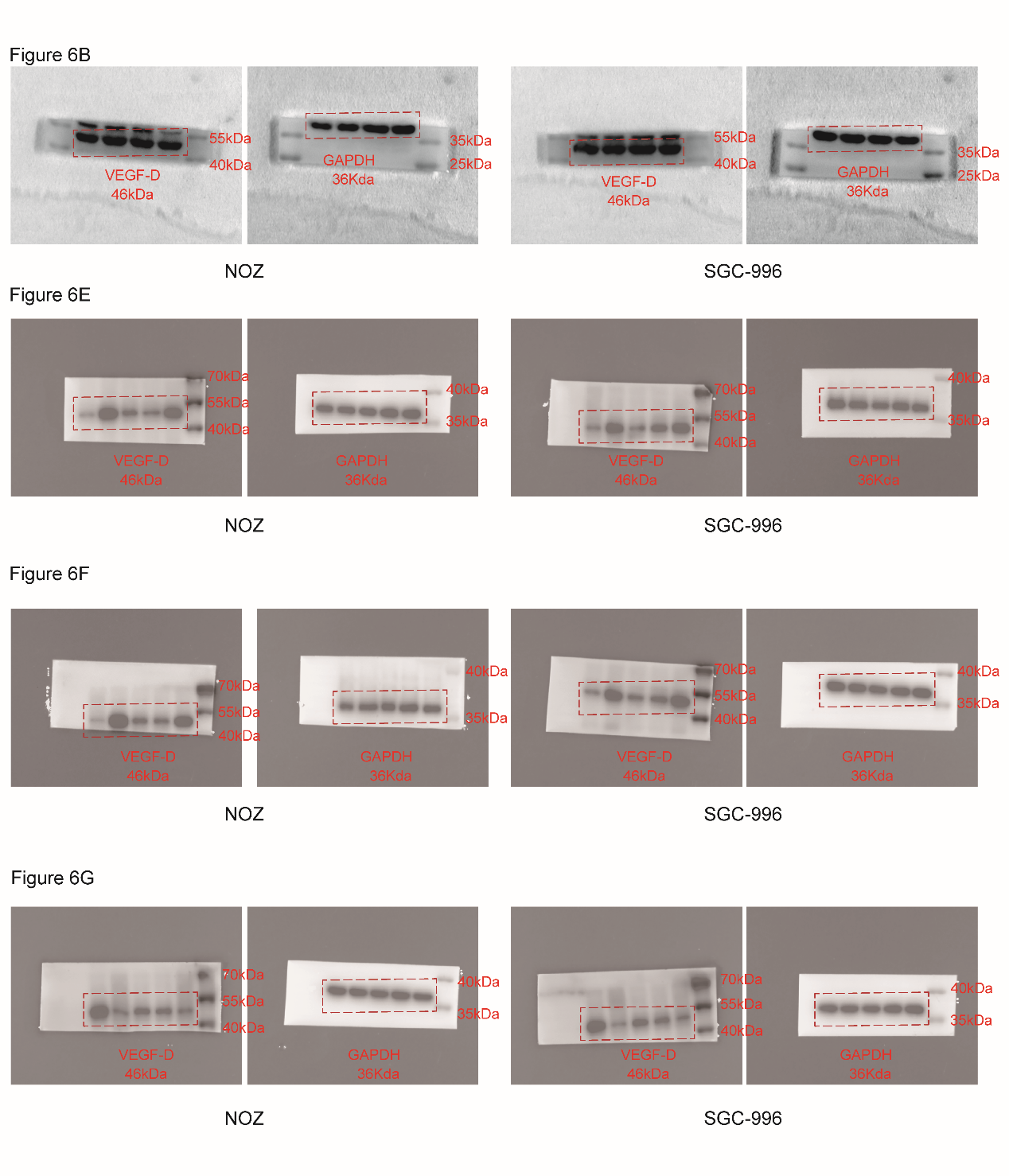


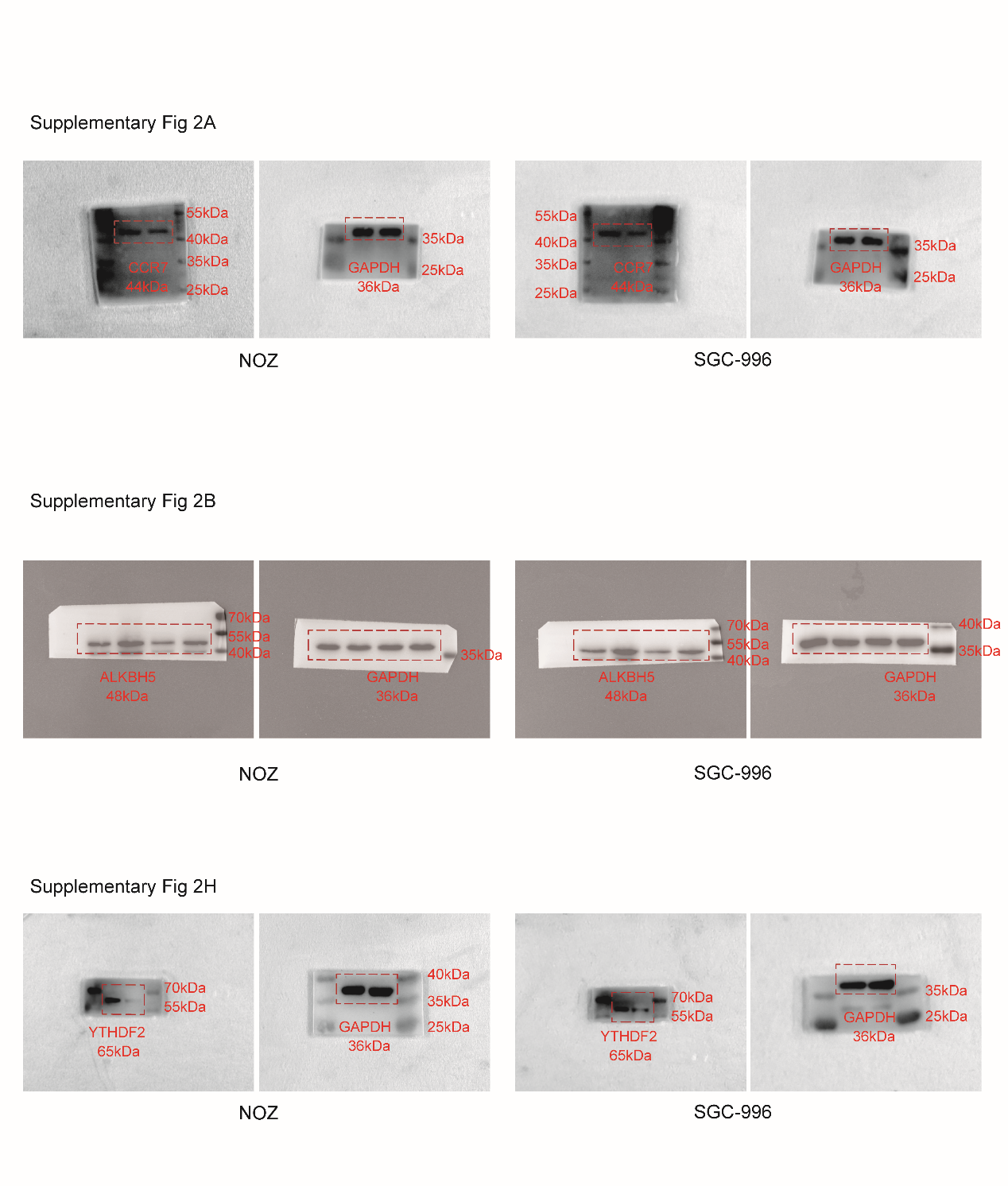

Supplement: Supplementary file 1 — Supplementary file1 (DOCX 3781 KB) [file 13577_2026_1420_MOESM1_ESM.docx]

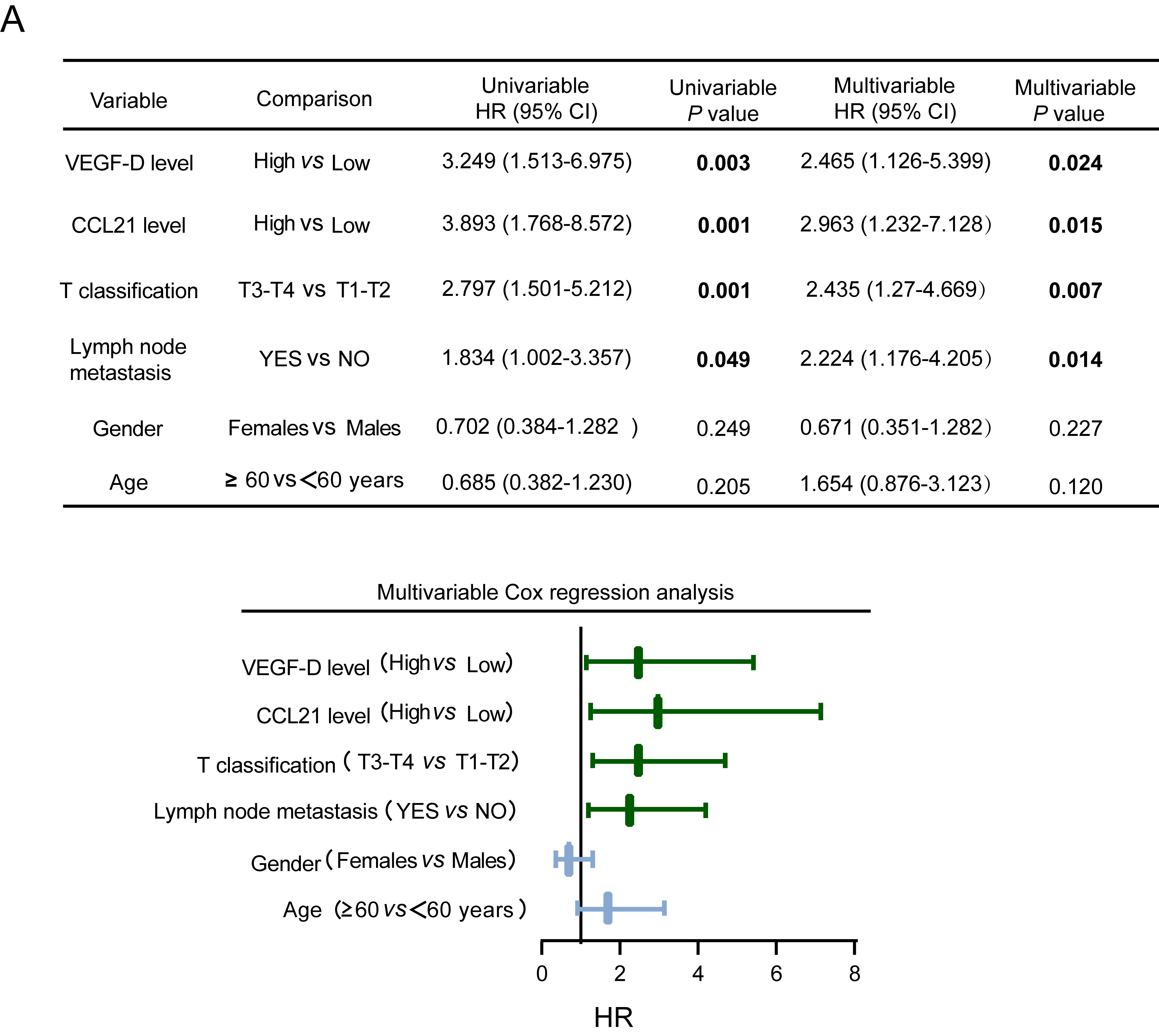

Supplement: Supplementary file 2 — Figure S1: CCL21/CCR7 promotes GBC LNM via VEGF-D. (A) The upper panel shows the univariable and multivariable Cox regression analyses of factors associated with overall survival in patients with GBC, including comparison groups, hazard ratios (HRs), 95% confidence intervals (CIs), and exact P values. The lower panel shows the forest plot of the multivariable Cox regression analysis. The Cox regression analysis included 65 patients with available survival data, including 47 death events. Variables entered into the multivariable model included VEGF-D level, CCL21 level, T classification, lymph node metastasis, age, and gender. Bold values indicate P < 0.05. Supplementary file2 (TIF 2426 KB) [file 13577_2026_1420_MOESM2_ESM.tif]

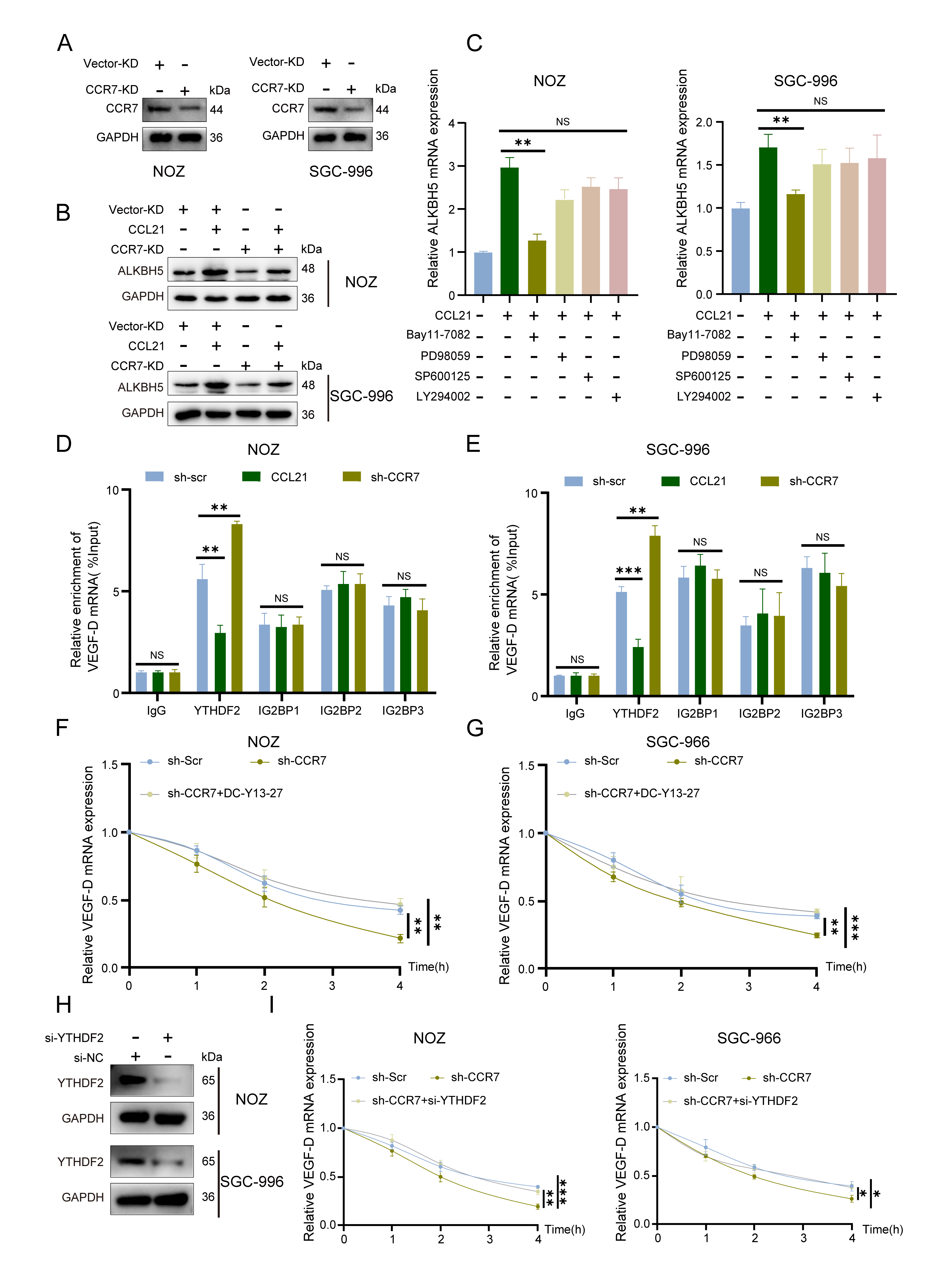

Supplement: Supplementary file 3 — Figure S2: CCL21/CCR7 inhibits m6A modification of VEGF-D mRNA via ALKBH5 in GBC cells. (A) Validation of CCR7 knockdown efficiency by CRISPR–Cas9 (CCR7-KD). (B) Rescue experiments showing the effect of CCR7-KD on CCL21-mediated ALKBH5 expression by Western blot. (C) RT-qPCR analysis of the inhibitory effects of pathway inhibitors on CCL21-induced ALKBH5 mRNA expression. (D-E) RIP-qPCR assays demonstrating the binding of YTHDF2 and IGF2BPs to VEGF-D mRNA. (F-G) YTHDF2 inhibitor treatment increased VEGF-D mRNA stability in CCR7-knockdown cells. (H) Western blot analysis confirming the knockdown efficiency of si-YTHDF2 in GBC cells. (I) Knockdown of YTHDF2 by siRNA partially rescued the decrease in VEGF-D mRNA stability induced by CCR7 knockdown. Error bars represent the mean（n=3）±SEM. **P＜0.01, ***P＜0.001. Supplementary file3 (TIF 5050 KB) [file 13577_2026_1420_MOESM3_ESM.tif]

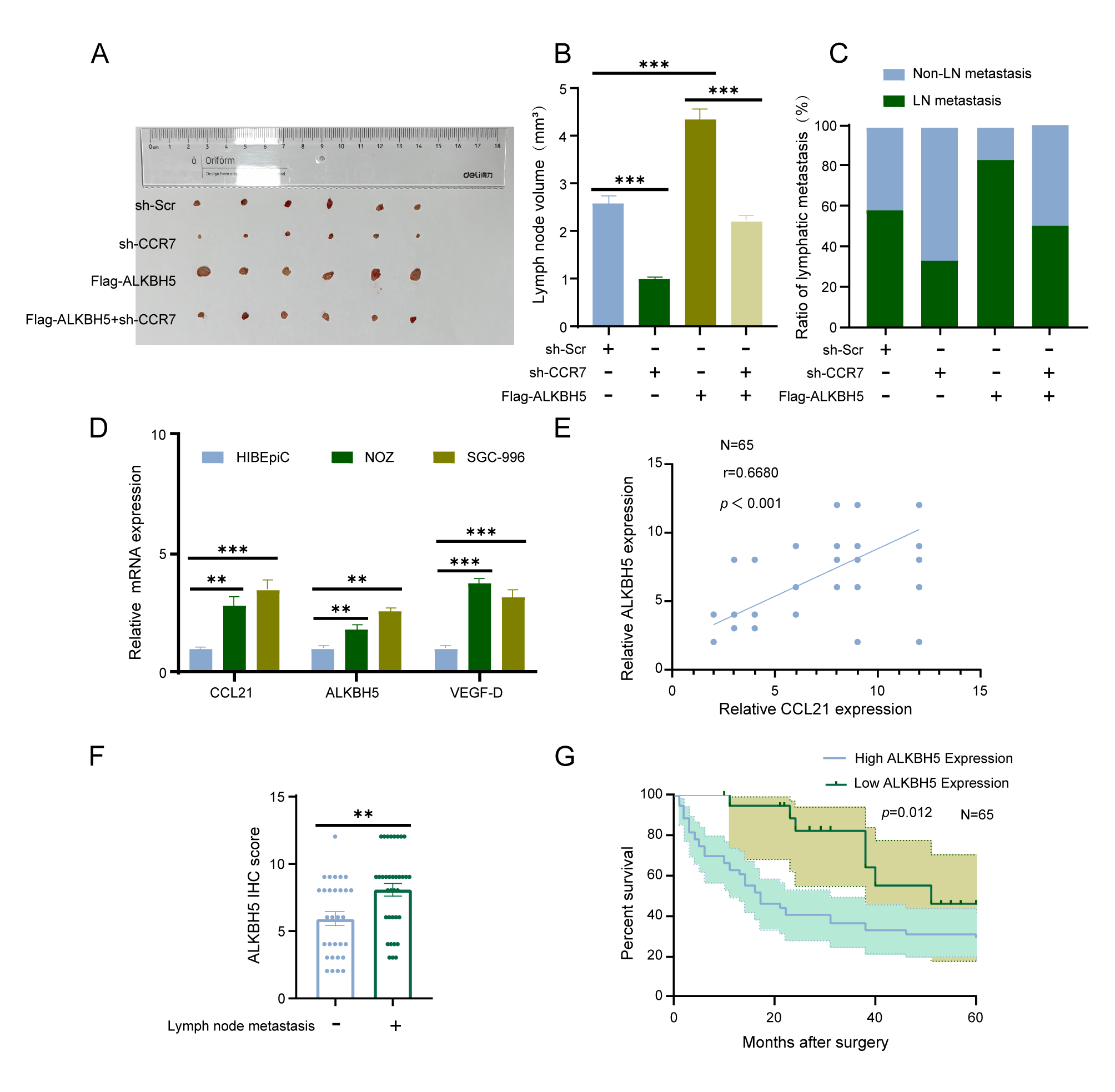

Supplement: Supplementary file 4 — Figure S3: ALKBH5 promotes lymphatic metastasis and is clinically associated with CCL21/CCR7 signaling in GBC. (A-C) Effects of Flag-ALKBH5 overexpression on CCL21/CCR7 axis-mediated lymphatic metastasis in nude mice. (A) Lymph node size, (B) lymph node volume, and (C) lymph node metastasis rate. (D) mRNA expression levels of CCL21, ALKBH5, and VEGF-D in normal biliary epithelial cells and GBC cell lines. (E) Correlation analysis of CCL21 and ALKBH5 expression in GBC tissues based on IHC from 65 patients. (F) Comparison of ALKBH5 expression between GBC patients with and without lymph node metastasis. (G) Kaplan-Meier survival analysis of GBC patients stratified by ALKBH5 expression. Error bars represent the mean（n=3）±SEM. **P＜0.01, ***P＜0.001. Supplementary file4 (TIF 1771 KB) [file 13577_2026_1420_MOESM4_ESM.tif]
